# A Raman algorithm to estimate human age from protein structural variations in autopsy skin samples: a protein biological clock

**DOI:** 10.1038/s41598-021-85371-7

**Published:** 2021-03-15

**Authors:** Daisuke Miyamori, Takeshi Uemura, Wenliang Zhu, Kei Fujikawa, Takaaki Nakaya, Satoshi Teramukai, Giuseppe Pezzotti, Hiroshi Ikegaya

**Affiliations:** 1 Department of Forensic Medicine, Graduate School of Medicine, Kyoto Prefectural University of Medicine, Kamigyo-ku, 465 Kajii-cho, Kawaramachi dori, Kyoto, 602-0841 Japan; 2 Ceramic Physics Laboratory, Kyoto Institute of Technology, Sakyo-ku, Matsugasaki, Kyoto 606-8126 Japan; 3 Department of Biostatistics, Graduate School of Medicine, Kyoto Prefectural University of Medicine, Kamigyo-ku, 465 Kajii-cho, Kawaramachi dori, Kyoto, 602-0841 Japan; 4 Department of Infectious Diseases, Graduate School of Medicine, Kyoto Prefectural University of Medicine, Kamigyo-ku, 465 Kajii-cho, Kawaramachi dori, Kyoto, 602-0841 Japan

**Keywords:** Biomarkers, Geriatrics, Microscopy

## Abstract

The recent increase of the number of unidentified cadavers has become a serious problem throughout the world. As a simple and objective method for age estimation, we attempted to utilize Raman spectrometry for forensic identification. Raman spectroscopy is an optical-based vibrational spectroscopic technique that provides detailed information regarding a sample’s molecular composition and structures. Building upon our previous proof-of-concept study, we measured the Raman spectra of abdominal skin samples from 132 autopsy cases and the protein-folding intensity ratio, R_PF_, defined as the ratio between the Raman signals from a random coil an α-helix. There was a strong negative correlation between age and R_PF_ with a Pearson correlation coefficient of r = 0.878. Four models, based on linear (R_PF_), squared (R_PF_^2^), sex, and R_PF_ by sex interaction terms, were examined. The results of cross validation suggested that the second model including linear and squared terms was the best model with the lowest root mean squared error (11.3 years of age) and the highest coefficient of determination (0.743). Our results indicate that the there was a high correlation between the age and R_PF_ and the Raman biological clock of protein folding can be used as a simple and objective forensic age estimation method for unidentified cadavers.

## Introduction

Recently, the number of the unidentified cadavers has increased due to disasters and terrorisms episodes worldwide, and as a consequence of social internationalization^1,2^.

Amongst the various types of information needed for cadaver identification, age is the most important. Most of the conventional age estimation methods, including anthropological methods and the dental method^3^ are mainly used in highly corrupted or skeletonized corpses. Closing of the cranial suture^4^, change of the pubic bone^5^, morphology of the ribs^6^, the developmental stages of the seven left permanent mandibular teeth^7^ and so on represent some examples of those methods. Some of the above-mentioned methods provide a high estimation accuracy^8–10^; however, they are mainly subjective and require extensive technical skills and experience^2,11–13^. Recently, computed tomography (CT) and magnetic resonance imaging (MRI) have also been used in this area^14–17^. CT and MRI require very expensive equipment, and therefore their use is not yet widely adopted.

Age estimation is somewhat thought to be necessary only for highly corrupted corpses, but fresh corpses that require age estimation are found on a daily basis. For age estimation in fresh cadavers or living individuals, in addition to above mentioned methods, bone X-ray examination, physical examination, dental eruptions, etc.^18^, molecular biological methods are also used. For instance, among the alterations that occur to our body as we age, many relate to proteins, and attach to intrinsic variations of their molecular structure. Stadtman^19^ proved how the age-related increase in oxidized proteins might reflect the age-dependent accumulation of unrepaired DNA damage.

The most advanced and objective concept of an “age predictor” in modern forensic science matches the so-called epigenetic (or DNA, or Horvath’s) clock^20–22^, which is based on the DNA methylation of CpG dinucleotides and can be used to estimate the age of a suspect based on blood remains. Usable on a wide spectrum of different tissues, the median error in age prediction of this method is 3.6 y, with a Pearson correlation coefficient, r = 0.96, to chronological age^21^. The stronger the association of the two variables, the closer the Pearson correlation coefficient, r, with this coefficient being either + 1 or − 1 depending on whether the relationship is positive or negative, respectively. Horvath’s clock leads to a remarkably precise age estimation as compared to other proposed methods (e.g., telomere length, p16INK4a, or microsatellite mutations)^23–25^, which show distinctly lower correlation coefficients and only relate to specific types of cells rather than directly to the chronological age. The epigenetic clock was also found to relate to the body mass index (BMI) with a good correlation, r = 0.42, for analyses conducted on liver tissue^21^. Although Horvath’s method proved highly objective and has remarkable accuracy among the various age estimation methods, special technical skills, expensive reagents and equipment are still required as well as time and effort.

Looking for a different approach, we examined the possibility of using Raman spectroscopy of human skin in an attempt to establish a reliable and easily accessible method of age estimation in forensic medicine. Our spectroscopic method relies on the collection of incisional skin samples during forensic autopsy, and spectroscopic examination of the structural characteristics of both the proteins and lipids contained in the dermis (including epidermis, dermis, and hypodermis). Aging of the structures of skin has been deeply studied by employing a number of different analytical techniques^26,27^. Our previously published proof-of-concept study attempted to label all the skin-emitted Raman bands and to locate any biophysical link between vibrational fingerprints and specific structural variations associated with human age^28^. The existence of such links was indeed phenomenologically proven for protein structures; however, it is hindered by insufficient statistics. The final proof for our spectroscopic findings, thus, relies on the statistical validations.

The main purpose here is a statistical validation of the presence of Raman spectral markers for precise age identification from human skin samples. In doing so, we used the same spectral deconvolution algorithm previously proposed^28^. In that previous study, we investigated the Raman spectra of various skin samples to find a candidate of ideal structural change for age estimation with a small number of cases. Accordingly, we found that protein folding may be a good potential “biological clock”. In this study, upon increasing the number of autopsy samples (i.e., n = 132) and, concurrently, taking care that a wide age range was included, we attempted to assess the statistical exactness of age estimation by Raman spectrometry. The statistical validation presented here could be attractive for the forensic community because the Raman method is label-free and directly links vibrational features at the molecular scale to practical forensic purposes.

**Figure 1:**
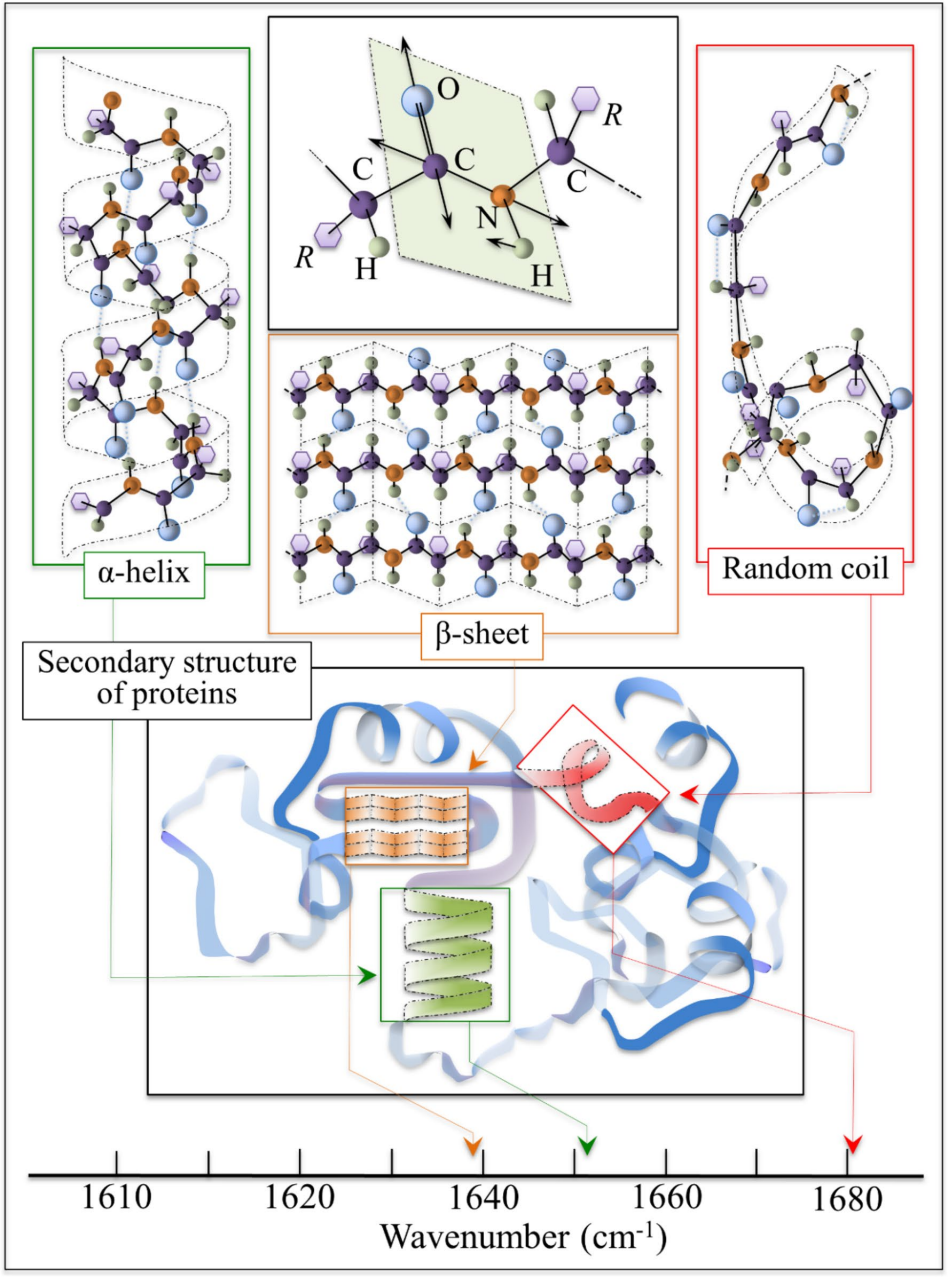
Explanatory schema of the different secondary structures involved with the spectroscopic measurement of human age based on the protein folding mechanism. Three kinds of protein mesostructures (shown in Table S2) are detected by Raman spectroscopy. Our previous study^28^ suggested that the rate of random coiled disordered protein structures (Raman signal seen at 1681 cm^−1^) becoming α-helix structures (Raman signal at 1652 cm^−1^) was age dependent.

Figure 1 gives schematic drafts of the different secondary structures of proteins detected by Raman spectroscopy. Figure 1 also includes drafts of the specific vibrational modes that were monitored in this study, as well as the Raman spectroscopic links in terms of the vibrational frequencies of different structural assemblies. The structure of skin upon aging has been thoroughly studied with a number of different analytical techniques^26,27^. Unfortunately, explicit biophysical links are still missing between the observed vibrational fingerprints and the specific structural variations associated with human age. With reference to the relative intensities of specific Raman scatterings from different protein mesostructures (α-helix structure and disordered random coil protein structures as seen in Fig. 1), we defined the protein-folding intensity ratio (R_PF_). The R_PF_ represents the ratio of Raman intensities related to the C=O stretching mode (i.e., in the overall Amide I vibrational spectrum depicted in Table S2) in disordered protein structures (i.e., the random coil Raman signal seen at 1681 cm^−1^) to α-helix structure (i.e., the Raman signal at 1652 cm^−1^) (Fig. 1)^29–37^.

Studies^29,30^ have reported that proteins irreversibly fold upon aging. Accordingly, in our previous study^28^, the Raman features representing those folding processes were located as parameters sensing biological age, although their age-sensing precision was yet unknown to us.

The aim of this study was to statistically validate the Raman spectroscopic parameters of protein folding in human skin as age-predicting parameters. The Raman spectra of abdominal skin sample were measured in 132 autopsy cases and the protein-folding intensity ratio (R_PF_) was calculated. And four models, based on linear (R_PF_), squared (R_PF_^2^), sex, and R_PF_ by sex interaction terms, were examined.

## Results

**Figure 2:**
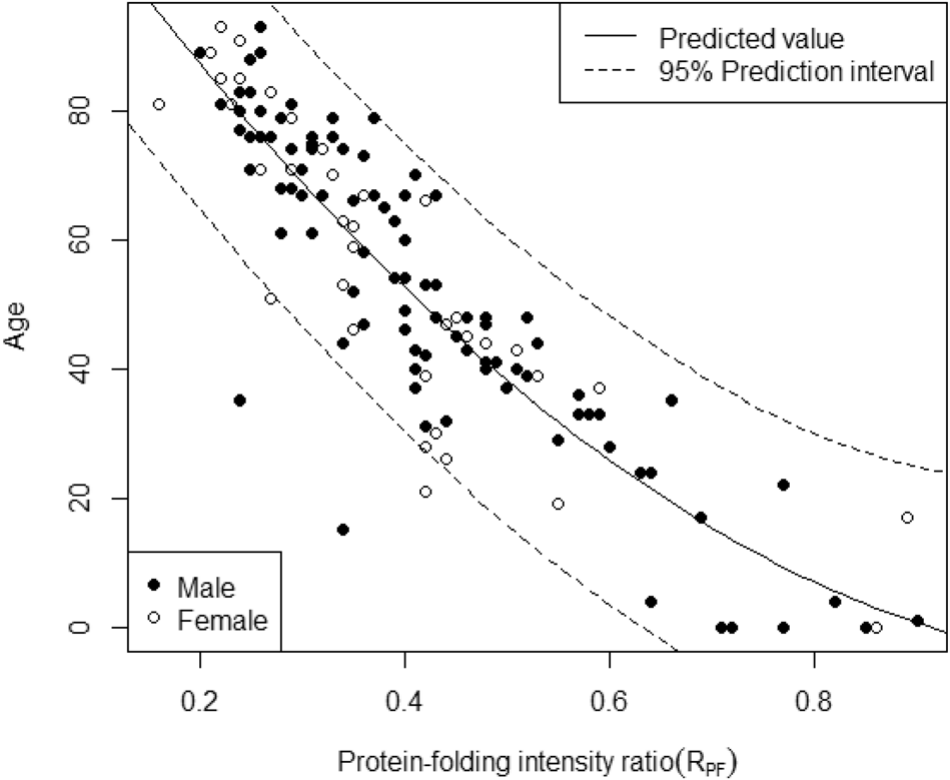
Prediction curve using Model 2 (R_PF_). Complete plots for all age intervals are given for the protein-folding intensity ratio (R_PF_) and the 95% prediction intervals are also shown. There was a strong negative correlation between age and the R_PF_ with a Pearson correlation coefficient of r = 0.878.

The data from a total of 132 subjects (92 males and 40 females) were analyzed. Table 1 shows the summary statistics of age and R_PF_. The R_PF_ of our samples ranged from 0.16 to 0.90. The mean years of age and R_PF_ were 53.4 and 0.41, respectively. On the other hand, the median years of age and R_PF_ were 53.5 and 0.39, respectively. The estimated regression coefficients for four models were shown in Table 2. The results of cross validation suggested that Model 2 including linear and squared terms was the best model with the lowest root mean squared error (11.3 years of age) and the highest coefficient of determination (0.743). Both estimates for R_PF_ and R_PF_^2^ were statistically significant at the 5% significance level in Model 2. There was a strong negative correlation between age and R_PF_ with a Pearson correlation coefficient of r = − 0.878. Complete plots for all age intervals are given in Fig. 2 for the R_PF_. Figure 2 also shows the predicted values of age and the 95% prediction intervals. Table 3 shows the 95%, 80%, and 50% prediction intervals. For instance, if the R_PF_ is 0.3, the 95%, 80%, and 50% prediction intervals are 46.7–91.3 years of age, 54.5–83.5 years of age and 61.4–76.6 years of age, respectively. If the R_PF_ would be 0.5, the 95%, 80%, and 50% prediction intervals are 15.9–60.6 years of age, 23.7–52.8 years of age and 30.6–45.9 years of age, respectively. There was no statistically significant correlation between the R_PF_ and BMI. There was no statistically significant difference between the male and female samples. Moreover, the specific cause of death was not seen in the outliers.

**Table 1:** Descriptive statistics for age and R_PF_.

	Age	
Mean	53.4	0.41
Standard deviation	24.2	0.16
Median	53.5	0.39
Range	0–93	0.16–0.90

n = 132.

R_PF_: protein-folding intensity.

**Table 2:** The regression formulae used in this study.

Model	Regression formula
1	age = 108.4 − 133.8 × R_PF_
2	age = 130.0 − 233.3 × R_PF_ + 99.4 × R_PF_^2^
3	age = 129.3 − 237.2 × R_PF_ + 102.7 × R_PF_^2^ + 2.4 × sex (M = 1, F = 0)
4	age = 106.8 − 132.5 × R_PF_ + 2.7 × sex (M = 1, F = 0) − 2.5 × R_PF_・sex (M = 1, F = 0)

R_PF_: protein-folding intensity, R_PF_^2^: the square of R_PF_.

**Table 3:** R_PF_ and the age prediction intervals for 50, 80 and 95% by confidence levels.

R_PF_	Prediction interval (years old)		
	50%	80%	95%
0.3	61.4–76.6	54.5–83.5	46.7–91.3
0.4	45.0–60.2	38.1–67.1	30.4–74.9
0.5	30.6–45.9	23.7–52.8	15.9–60.6
0.6	18.2–33.5	11.3–40.4	3.5–48.2
0.7	7.7–23.1	0.7–30.1	0–37.9

R_PF:_ protein-folding intensity.

## Discussion

To quickly identify unidentified cadavers, age estimation methods that can be used by non-experts at the places where bodies are found must be developed Recently, biomarkers, such as prostate specific antigen and thyroid hormones, skin imaging, and ultrasonography have been reported as alternative age estimation methods to the conventional subjective methods^38–42^. Skin imaging and ultrasonography are particularly elementary and can be used by non-experts on the scene^38,41^. Unfortunately, their estimation accuracy is relatively low.

In this study, a large number of analytical data showed that R_PF_, related to the folding in peptides and proteins, was a reliable marker for the estimation of human age. The reason for the observed precision of the age-predictor, R_PF_, might reside in the fact that protein oxidation and misfolding mainly affect the frequency shift of the sub-bands used for age assessment, while they minimally alter the Raman intensity. Although the outputs of this study were validated with a statistically analyzable number of patients and the shown equations are presently used at the Kyoto Prefectural University of Medicine for estimating the age of unidentifiable bodies, our presented method still requires a substantial amount of time and skill.

Recently, hand-held Raman spectrometric devices were developed^43^. This device is now available in each prefectural police department in Japan for drug detection at crime scenes and requires less than a minute for detection, no expensive reagents, and technical skills. Considering our results, this equipment appears a promising choice for age estimation of unidentified cadavers in instantaneous assessments at the crime scene by simply detecting R_PF_ related bands.

There are certain limitations in our study. First, although there was no statistically significant difference between the male and female samples, this may be related to our sample size. Therefore, we need to continue increasing the sample number. Second, although the specific causes of death were not related to the outliers, we could not perform a statistical analysis due to the diverse causes of death. Third, the confidence intervals of age prediction were relatively wide especially in young people. Many methods that use growing and development characteristics are reported as reliable for individuals under 20 years of age^44,45^.

Therefore, we consider that age estimation should be done by the combination of many methods to obtain better accuracy and avoid erroneous judgments. Finally, our sampling was restricted to Japanese donors living in the same area and climate; nevertheless the results are evocative and must next be confirmed with future experiments regarding whether (or to what extent) the Raman biological clock of protein folding is common to all human beings.

## Materials and methods

### Materials

A total of 132 cadaveric skin samples were obtained from forensic autopsies for cadavers at different ages spanning from a few months to 93 y old (Table S1 in the Supplementary Materials). Relatives of all the subjects gave their informed consent before inclusion in the study. The study was conducted in accordance with the Declaration of Helsinki, and the protocol was approved by the Institutional Review Board of Kyoto Prefectural University of Medicine, Japan (ERB C-191). Samples were taken during the autopsies conducted within 48 h.

Several samples for each patient were taken from the sub-umbilical region of abdominal skin in the body, which typically remains unexposed to solar irradiation. The sampling included only Japanese subjects. A list of the donors with information on their age, height and weight at death, sex is given in Table S1. In the statistical characterization, no distinction was made regarding the sampling; therefore, we included in the Raman analysis any available sample irrespective of the cause of death. The skin samples were 10 × 10 mm^2^, and encompassed the full thickness of the skin from the stratum corneum to the hypodermis.

### Spectroscopy

**Figure 3:**
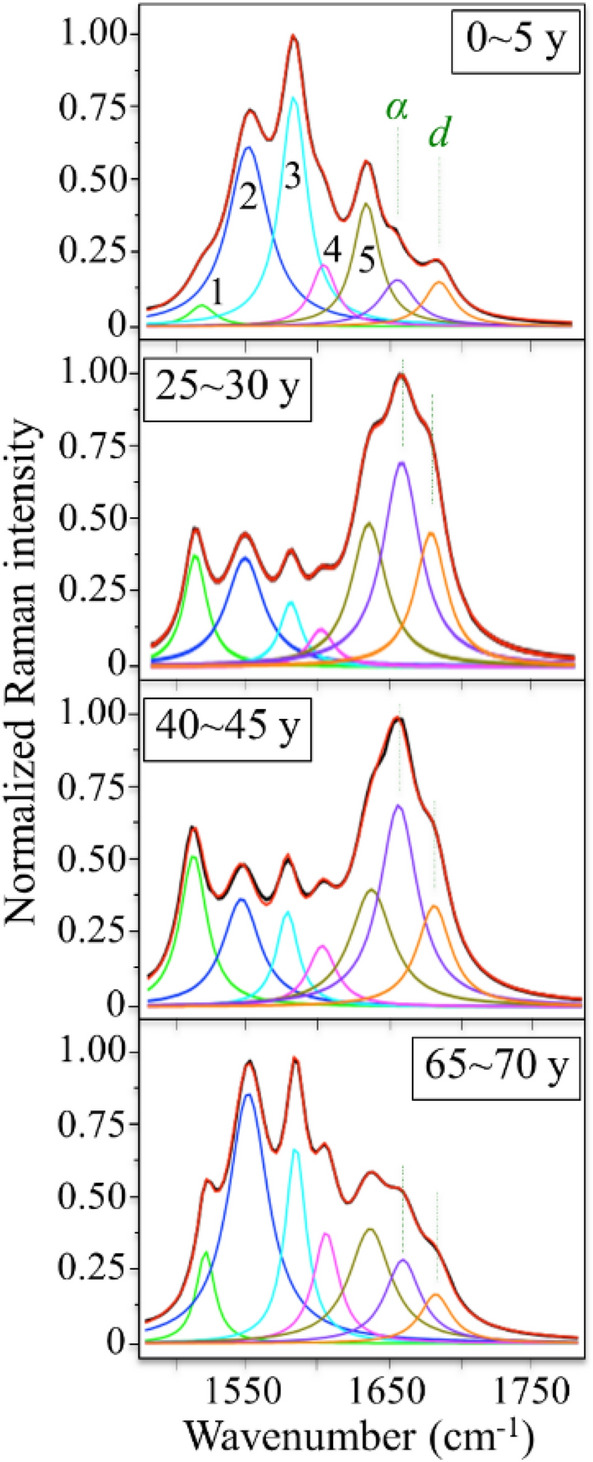
Average Raman spectra from skin samples from the patients listed in Table S1 for the selected age intervals (shown in inset). Spectroscopic related to the proteins are shown. In the protein spectral zone, the labels α and d refer to α-helix and disordered structures, respectively. The vibrational origins of all labeled bands are listed in Table S2 of the Supplementary Materials and are visualized in Fig. 1.

The samples were stored in a humid and evacuated environment at approximately 0 °C until use. Raman spectroscopy was conducted within 5 days after the autopsy. The Raman excitation source in this experiments used a 532 nm Nd:YVO4 diode-pumped solid state laser operating with a power of 200 mW (SOC JUNO, Showa Optronics Co. Ltd., Tokyo, Japan). An objective lens with a numerical aperture of 0.5 was used both to focus the laser beam on the sample surface and to collect the scattered Raman light. A pinhole aperture of 100 μm was adopted employing an objective lens with a magnification of 100x. For each studied sample, several tens of spectra were collected from the epidermis to dermis (100–600 µm depth from the skin surface) in the cross-section and we obtained an average spectrum whose sub-bands were best-fitted to Gaussian–Lorentzian bells^46^. Table S2 shows a complete description of the sub-bands labeled in Fig. 3 for the protein spectral zone, which includes the frequency range at which these specific sub-bands were located and their vibrational origins. To have a reliable sampling of the bands per each donor, a number of Raman spectra in the order of > 100 needed to be averaged. Although the spectral acquisition procedure could be automatized, scanning of wide spectral range through the dermis to epidermis resulted in time consuming (about 20 h of measurement per donor).

### Statistics

We evaluated the correlation between age or the BMI and the R_PF_ using the Pearson correlation coefficient. To build a prediction model, we performed regression analysis with age as the dependent variable for four candidate models. The independent variables of those models were follows: R_PF_ in Model 1; R_PF_ and R_PF_^2^ (the square of R_PF_) in Model 2; R_PF_ , R_PF_^2^ and sex in Model 3; and R_PF_, sex, and R_PF_ by sex interaction in Model 4. The goodness-of-fit of each model was assessed using a tenfold cross validation method (repeated 100 times), estimating the mean squared error and coefficient of determination. After selecting a best model, we estimated 95% prediction intervals for the predicted ages.

## Supplementary Information

Supplementary Information.

## Data Availability

The datasets generated during and/or analysed during the current study are available from the corresponding author on reasonable request.
