# Supplementary material for: A Raman algorithm to estimate human age from protein structural variations in autopsy skin samples: a protein biological clock
Source: Sci Rep. 2021 Mar 15;11:5949. doi: 10.1038/s41598-021-85371-7 (PMC7960715; doi:10.1038/s41598-021-85371-7)
Supplement: Supplementary file 1 — Supplementary Information. [file 41598_2021_85371_MOESM1_ESM.docx]

Article

**A Raman algorithm to estimate human age from protein structural variations in autopsy skin samples: a protein biological clock**

Daisuke Miyamori,^1^ Takeshi Uemura,^1^ Wenliang Zhu,^2^ Kei Fujikawa,^3^ Takaaki Nakaya^4^, Satoshi Teramukai,^3*^ Giuseppe Pezzotti,^2*^ and Hiroshi Ikegaya ^1*^

1 Department of Forensic Medicine, Graduate School of Medicine, Kyoto Prefectural University of Medicine, Kamigyo-ku, 465 Kajii-cho, Kawaramachi dori, Kyoto 602-0841, Japan; [md199090@gmail.com](mailto:md199090@gmail.com), [uemura@amine-pharma.com](mailto:uemura@amine-pharma.com), [Ikegaya-tky@umin.ac.jp](mailto:Ikegaya-tky@umin.ac.jp)

2 Ceramic Physics Laboratory, Kyoto Institute of Technology, Sakyo-ku, Matsugasaki, Kyoto 606-8126, Japan; [wenlzhu@hotmail.com](mailto:wenlzhu@hotmail.com), [pezzotti@kit.ac.jp](mailto:pezzotti@kit.ac.jp)

3 Department of Biostatistics, Graduate School of Medicine, Kyoto Prefectural University of Medicine, Kamigyo-ku, 465 Kajii-cho, Kawaramachi dori, Kyoto 602-0841, Japan; [steramu@koto.kpu-m.ac.jp](mailto:steramu@koto.kpu-m.ac.jp), [kfuji13@koto.kpu-m.ac.jp](mailto:kfuji13@koto.kpu-m.ac.jp)

Department of Biostatistics, Graduate School of Medicine, Kyoto Prefectural University of Medicine, Kamigyo-ku, 465 Kajii-cho, Kawaramachi dori, Kyoto 602-0841, Japan; [steramu@koto.kpu-m.ac.jp](mailto:steramu@koto.kpu-m.ac.jp), [kfuji13@koto.kpu-m.ac.jp](mailto:kfuji13@koto.kpu-m.ac.jp)

4 Department of Infectious Diseases, Graduate School of Medicine, Kyoto Prefectural University of Medicine, Kamigyo-ku, 465 Kajii-cho, Kawaramachi dori, Kyoto 602-0841, Japan; tnakaya@koto.kpu-m.ac.jp

*Correspondence: ikegaya-tky@umin.ac.jp; Tel. +81-75-251-5343 (H.I): pezzotti@kit.ac.jp; Tel. +81-75- 724-7014 (G.P.)

**Table S1 Subject profile and protein-folding intensity ratio (RPF) of the skin.**

| Sample No. | Age | Sex | Height  (cm) | Weight  (kg) | R_PF_±SD |
| --- | --- | --- | --- | --- | --- |
| 1 | 3m | F | 66 | 6.3 | 0.72±0.13 |
| 2 | 1m | M | 44 | 1.6 | 0.85±0.40 |
| 3 | 1m | M | 59 | 4.5 | 0.77±0.21 |
| 4 | 1m | F | 42 | 2.4 | 0.86±0.34 |
| 5 | 2m | M | 57 | 5 | 0.71±0.24 |
| 6 | 3m | M | 67.5 | 7.7 | 0.72±0.22 |
| 7 | 1y | M | 85 | 12.4 | 0.90±0.33 |
| 8 | 4y | M | 107 | 22.6 | 0.64±0.11 |
| 9 | 4y | M | 101 | 16.5 | 0.82±0.27 |
| 10 | 15y | M | 164 | 55.2 | 0.34±0.13 |
| 11 | 17y | F | 148 | 38.5 | 0.89±0.44 |
| 12 | 17y | M | 180 | 69.1 | 0.69±0.12 |
| 13 | 19y | F | 158 | 48.1 | 0.55±0.37 |
| 14 | 21y | F | 174 | 61.3 | 0.42±0.22 |
| 15 | 22y | M | 168 | 33.1 | 0.77±0.33 |
| 16 | 24y | M | 171 | 71.4 | 0.63±0.30 |
| 17 | 24y | M | 169 | 52.8 | 0.64±0.35 |
| 18 | 26y | F | 136 | 16.9 | 0.44±0.41 |
| 19 | 28y | M | 160 | 57.1 | 0.60±0.31 |
| 20 | 28y | F | 150 | 64.3 | 0.42±0.33 |
| 21 | 29y | M | 170 | 65.2 | 0.55±0.18 |
| 22 | 30y | F | 145 | 41.1 | 0.43±0.24 |
| 23 | 31y | M | 175 | 65.2 | 0.42±0.31 |
| 24 | 32y | M | 181 | 99 | 0.44±0.27 |
| 25 | 33y | M | 169 | 55.4 | 0.57±0.28 |
| 26 | 33y | M | 156 | 52.6 | 0.58±0.23 |
| 27 | 33y | M | 185 | 87.2 | 0.59±0.11 |
| 28 | 35y | M | 173 | 73 | 0.24±0.21 |
| 29 | 35y | M | 174 | 69 | 0.66±0.17 |
| 30 | 36y | M | 178 | 51.2 | 0.57±0.23 |
| 31 | 37y | M | 160 | 50.7 | 0.41±0.22 |
| 32 | 37y | M | 176 | 80.3 | 0.50±0.25 |
| 33 | 37y | F | 146 | 48 | 0.59±0.24 |
| 34 | 39y | M | 173 | 61.4 | 0.52±0.21 |
| 35 | 39y | F | 153 | 52.9 | 0.53±0.33 |
| 36 | 39y | F | 176 | 84.6 | 0.42±0.31 |
| 37 | 40y | M | 170 | 66.4 | 0.41±0.20 |
| 38 | 40y | M | 173 | 72 | 0.48±0.11 |
| 39 | 40y | M | 170 | 67.1 | 0.51±0.21 |
| 40 | 41y | M | 173 | 106.5 | 0.49±0.18 |
| 41 | 41y | M | 175 | 95.5 | 0.48±0.19 |
| 42 | 42y | M | 169 | 46.8 | 0.42±0.15 |
| 43 | 43y | M | 173 | 59.7 | 0.41±0.14 |
| 44 | 43y | M | 175 | 69.9 | 0.46±0.14 |
| 45 | 43y | F | 156 | 43 | 0.51±0.22 |
| 46 | 44y | M | 165 | 69.7 | 0.53±0.31 |
| 47 | 44y | F | 165 | 76.1 | 0.48±0.30 |
| 48 | 44y | M | 172 | 78.7 | 0.34±0.25 |
| 49 | 45y | M | 157 | 79.4 | 0.45±0.13 |
| 50 | 45y | F | 168 | 67 | 0.46±0.27 |
| 51 | 46y | M | 166 | 75 | 0.40±0.19 |
| 52 | 46y | F | 149 | 27.6 | 0.35±0.20 |
| 53 | 47y | M | 175 | 66.5 | 0.36±0.18 |
| 54 | 47y | F | 158 | 71.8 | 0.44±0.20 |
| 55 | 47y | M | 175 | 55.3 | 0.48±0.23 |
| 56 | 48y | M | 170 | 73.4 | 0.43±0.18 |
| 57 | 48y | F | 155 | 54.3 | 0.45±0.16 |
| 58 | 48y | M | 175.5 | 62.5 | 0.48±0.15 |
| 59 | 48y | M | 180 | 71.4 | 0.52±0.17 |
| 60 | 48y | M | 162 | 56.9 | 0.46±0.18 |
| 61 | 49y | M | 171 | 54.6 | 0.40±0.12 |
| 62 | 51y | F | 154 | 40.7 | 0.27±0.20 |
| 63 | 52y | M | 172 | 59.1 | 0.35±0.18 |
| 64 | 53y | F | 155 | 51.5 | 0.34±0.17 |
| 65 | 53y | M | 166 | 60.7 | 0.42±0.22 |
| 66 | 53y | M | 175 | 54.2 | 0.43±0.25 |
| 67 | 54y | M | 167 | 57.8 | 0.39±0.18 |
| 68 | 54y | M | 169 | 49 | 0.40±0.20 |
| 69 | 54y | F | 166 | 38.4 | 0.40±0.21 |
| 70 | 58y | M | 170 | 77.9 | 0.36±0.23 |
| 71 | 59y | F | 160 | 54.8 | 0.35±0.11 |
| 72 | 60y | M | 166.5 | 51.2 | 0.40±0.18 |
| 73 | 61y | M | 167 | 69.7 | 0.28±0.24 |
| 74 | 61y | M | 170 | 103 | 0.31±0.15 |
| 75 | 62y | F | 153 | 32 | 0.35±0.13 |
| 76 | 63y | F | 153 | 45.8 | 0.34±0.21 |
| 77 | 63y | M | 156 | 65.5 | 0.39±0.22 |
| 78 | 65y | M | 153 | 80.2 | 0.38±0.25 |
| 79 | 66y | F | 150 | 45 | 0.42±0.26 |
| 80 | 66y | M | 158 | 53.6 | 0.35±0.21 |
| 81 | 67y | M | 154 | 58.6 | 0.30±0.21 |
| 82 | 67y | M | 154 | 50.5 | 0.32±0.18 |
| 83 | 67y | M | 167 | 99.4 | 0.37±0.16 |
| 84 | 67y | M | 157 | 49 | 0.40±0.18 |
| 85 | 67y | F | 148 | 35.3 | 0.36±0.12 |
| 86 | 67y | M | 170 | 73.7 | 0.43±0.18 |
| 87 | 68y | M | 175 | 90.8 | 0.28±0.20 |
| 88 | 68y | F | 159 | 59.5 | 0.29±0.15 |
| 89 | 68y | M | 168 | 83 | 0.29±0.16 |
| 90 | 70y | F | 155 | 68.6 | 0.33±0.18 |
| 91 | 70y | M | 164 | 35.1 | 0.41±0.17 |
| 92 | 71y | F | 152 | 47.9 | 0.29±0.17 |
| 93 | 71y | M | 167 | 49.7 | 0.30±0.21 |
| 94 | 71y | M | 163 | 67.4 | 0.25±0.18 |
| 95 | 71y | F | 155 | 43.2 | 0.26±0.19 |
| 96 | 71y | M | 154 | 41.7 | 0.30±0.15 |
| 97 | 73y | M | 165 | 47.3 | 0.36±0.17 |
| 98 | 74y | M | 175 | 52.3 | 0.34±0.18 |
| 99 | 74y | F | 162 | 37.6 | 0.32±0.21 |
| 100 | 74y | M | 158 | 49.4 | 0.31±0.19 |
| 101 | 74y | M | 168 | 56.2 | 0.29±0.15 |
| 102 | 75y | M | 166 | 69.2 | 0.31±0.16 |
| 103 | 76y | M | 152 | 55.9 | 0.25±0.16 |
| 104 | 76y | M | 165 | 61.2 | 0.31±0.18 |
| 105 | 76y | M | 181 | 80.2 | 0.33±0.21 |
| 106 | 76y | F | 161 | 57.5 | 0.26±0.16 |
| 107 | 76y | M | 168 | 52.9 | 0.26±0.18 |
| 108 | 76y | M | 170 | 61.3 | 0.27±0.20 |
| 109 | 77y | M | 155 | 42 | 0.24±0.14 |
| 110 | 79y | M | 155 | 31 | 0.33±0.22 |
| 111 | 79y | F | 152 | 45.4 | 0.29±0.17 |
| 112 | 79y | M | 154 | 53.8 | 0.28±0.18 |
| 113 | 79y | M | 166 | 56.6 | 0.37±0.19 |
| 114 | 80y | M | 160 | 46.6 | 0.24±0.16 |
| 115 | 80y | M | 157 | 56.9 | 0.26±0.18 |
| 116 | 81y | M | 166 | 64.5 | 0.29±0.16 |
| 117 | 81y | F | 141 | 53.8 | 0.16±0.11 |
| 118 | 81y | M | 160 | 58.2 | 0.22±0.12 |
| 119 | 81y | F | 143 | 33.1 | 0.23±0.13 |
| 120 | 83y | M | 165 | 34.5 | 0.24±0.16 |
| 121 | 83y | M | 169 | 51.8 | 0.25±0.17 |
| 122 | 83y | M | 162 | 62.4 | 0.24±0.16 |
| 123 | 83y | F | 163 | 38.5 | 0.27±0.15 |
| 124 | 85y | F | 155 | 43.5 | 0.24±0.12 |
| 125 | 85y | F | 156 | 41.6 | 0.22±0.11 |
| 126 | 88y | M | 157 | 50.4 | 0.25±0.15 |
| 127 | 89y | M | 170 | 55.4 | 0.26±0.13 |
| 128 | 89y | M | 160 | 63.7 | 0.20±0.11 |
| 129 | 89y | F | 145 | 34.4 | 0.21±0.12 |
| 130 | 91y | F | 139 | 34.2 | 0.24±0.12 |
| 131 | 93y | M | 165 | 46 | 0.26±0.12 |
| 132 | 93y | F | 153 | 41.8 | 0.22±0.10 |

m: month, y:year, M: male, F: female

**Table S2 Vibrational origins of all labeled bands in the protein spectral zone.**

| Band label | Band position (cm-1) | Principal assignment |
| --- | --- | --- |
| Band 1 | 510 | v(S-S) in cysteine |
| Band 2 | 745 | Symmetric breathing of tryptophan |
| Band 3 | 877 | v(C-C) in hydroxyproline, δ(tryptophan ring) |
| Band 4 | 919 | v(C-C) of proline rings |
| Band 5 | 1003 | v(C-C) in phenylalanine |
| Band 6 | 1081 | v(CO32-) and v(PO43-), v(C-C) in lipids |
| Band 7 | 1157 | Sphingomyelin |
| Band 8 | 1208 | v(C-C6H5) in tryptophan and phenylalanine |
| Band A- | 1210 | v(C-C6H5) in tyrosine and phenylalanine |
| Band A | 1218 | Amide III, v(C-N) and δ(N-H) in α-helix |
| Band 1* | 1242 | Amide III, v(C-N) and w(CH2) in collagen |
| Band 9 | 1255, 1258 | Amide III, adenine and cytosine β-sheet structure + lipids |
| Band 10 | 1271 | Non-polar fragments of proline in α-helix |
| Band 3* | 1315 | Amide III, δ(CH2) in α-helix collagen |
| Band B | 1338 | Amide III, v(C-N) and δ(N-H) in hydrated α-helix |
| Band C | 1370 | Ring and v(C-N) in cytosine and guanine |
| Band D | 1395 | Symmetric δ(CH3) of the methyl groups of proteins |
| Band 11A | 1428 | Proteins + lipids, CH2 scissoring |
| Band 11B | 1450 | δ(CH) in proteins + lipids |
| Band 11C | 1467 | δ(CH2) in proteins + lipids |
| Band 12 | 1526 | Sphingomyelin |
| Band E | 1520 | v(C=N) in adenine and cytosine in α-helix |
| Band F | 1551 | Amide II, v(C-N) and δ(N-H) |
| Band G | 1584 | δ(C=C) in phenylalanine |
| Band H | 1605 | v(C=C) in phenylalanine and tyrosine |
| Band I | 1638 | Amide I, v(C=O) in α-helix + β-sheet |
| Band 13 | 1652 | Amide I, v(C=O) in α-helix |
| Band J | 1681 | Amide I, v(C=O) in disordered structure |
| Band 14 | 1750 | v(C=O) in lipids and phospholipids |
